# Slow maxillary expansion in adult patient with Hyrax expander: A case report

**DOI:** 10.4317/jced.62001

**Published:** 2024-09-01

**Authors:** Benjamin Hostetter, Karthikeyan Subramani

**Affiliations:** 1 Roseman University of Health Sciences, College of Dental Medicine, Henderson, NV, USA

## Abstract

Slow maxillary expansion is a technique used to achieve correction of maxillary transverse deficiency or posterior crossbite in patients where the midpalatal suture has closed. This is mostly achieved by buccal tipping of maxillary posterior teeth. The aim of this case report is to discuss the orthodontic treatment of a 20-year-old patient with bilateral posterior crossbite. The patient had moderate maxillary crowding and severe mandibular crowding, crossbites bilaterally on his posterior teeth, and maxillary lateral incisors. The patient had thin gingival biotype with gingival recession on the mandibular right canine. Orthodontic treatment was done with full fixed appliances, and extraction of a mandibular right lateral incisor. This case report shows that slow maxillary expansion can be used in an adult to achieve the objectives set by both the orthodontist and patient while also considering treatment modalities most agreeable to the patient.

** Key words:**Orthodontic treatment, slow maxillary expansion, maxillary expansion, RPE, Hyrax expander, case report.

## Introduction

Rapid Palatal Expansion (RPE) is a commonly used treatment modality to resolve maxillary transverse discrepancy in patients whose mid-palatal suture has not yet fused (1). When patients do not get treated for maxillary transverse discrepancy, it persists into adulthood, at which point the patient may require surgically assisted RPE (SARPE) or mini-implant assisted RPE (MARPE). If rapid palatal expander is used after the mid-palatal suture has fused, sutural separation may be minimal and the transverse expansion is mainly achieved through buccal tipping of the maxillary posterior teeth (2,3). It has been reported in the literature that the midpalatal suture fuses at age 20 years in females and 25 years in males (4,5). Contrarily, it has also been reported that the palatal suture closes as early as 12-13 years of age (6). With varied reports on timing of sutural closure and fusion, the purpose of this case report was to use a Hyrax Rapid Palatal Expander with slow maxillary expansion (SME) protocol to correct a bilateral posterior crossbite in an adult patient. This is with the understanding from the literature that midpalatal sutural opening in an adult is unlikely and this treatment modality will most likely result in buccal tipping of posterior teeth to correct the posterior crossbite.

The expansion protocol for SME has been described as 1 turn every other day, with expansion maintenance for 12 weeks (7). The Hyrax expander is a tooth borne appliance attached with bands to first molars and first premolars on both sides of the maxillary arch. The expander is designed not make contact with the palate, which makes it easier to clean and therefore results in less irritation of the palatal mucosa (1).

## Case Report

-History

A Caucasian Male, 20 years 6 months of age, presented to the orthodontic clinic with chief complaint “I can visibly see the crowding and my teeth are not aligned”. A detailed dental, medical and social history was obtained from the patient. Patient had no contributory medical conditions reported. The patient had no known allergies and was not on any medications. The patient’s cervical vertebral maturation (CVMS) was stage V with no adolescent growth remaining.

-Assessment

**Figure 1:**
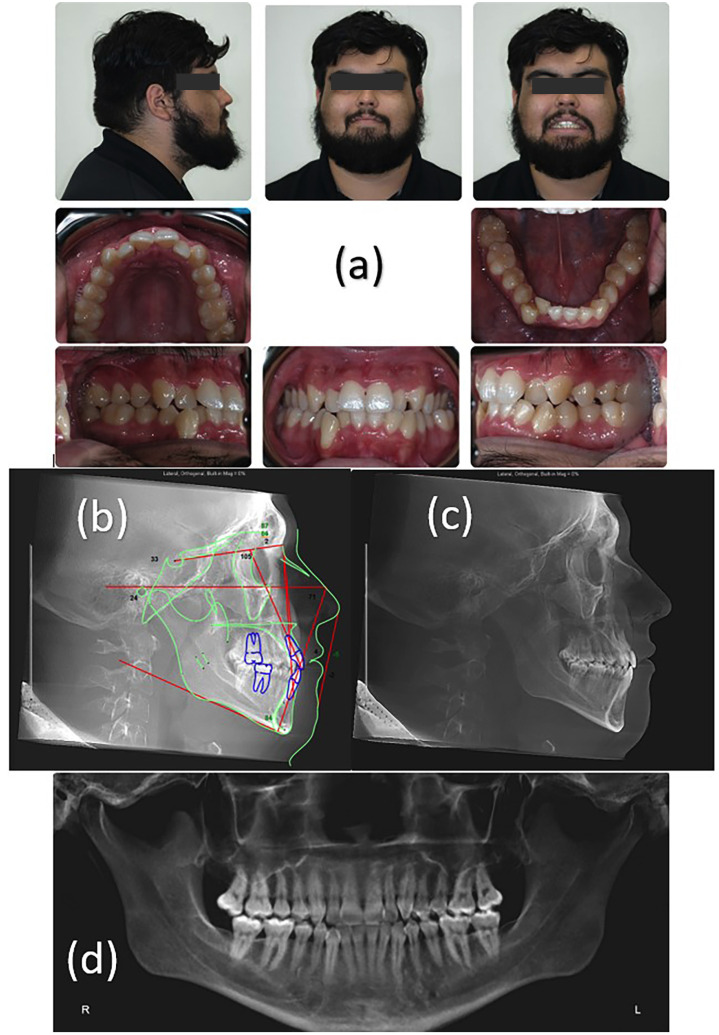
(a) Pretreatment facial and intraoral photographs, (b) Pretreatment lateral cephalogram, (c) tracing, (d) panoramic radiograph.

Clinical examination showed lip competence at repose, mildly convex facial profile, mesofacial, obtuse nasolabial angle, long lower facial third, and retruded upper lip. Patient had full step Class III molar and canine relationship bilaterally. Overbite and overjet were both measured to be 2 mm. Maxillary midline was 2 mm to the right of midsagittal plane and the mandibular midline was 1 mm to the right of midsagittal plane. In addition, there was 5-6 mm of maxillary crowding and 7-8 mm of net mandibular crowding. Posterior buccal crossbite was noted on the maxillary right and left second premolar, first molar, and second molars. Anterior crossbite was also noted on both maxillary lateral incisors. Gingival recession was noted on mandibular right canine with a generally thin gingival biotype. Occlusal cant was observed with the right side being lower, slanting up to the left. Patient had a Bolton discrepancy of 2.17 mm with suspected discrepancy being large mandibular incisors (Fig. 1a).

The lateral cephalometric radiograph analysis revealed that the patient was skeletal class III with an ANB = 1.5° due to a prognathic mandible (SNB = 85.5°). Patient also had a low mandibular plane angle (MP-FH = 24.36°) with no growth potential remaining. The maxillary incisors were within normal limits (U1-SN = 104.9°) and the mandibular incisors were retroclined (L1-MP = 84.4°). Upper (-4.6 mm) and lower (-1.9 mm) lips were retrusive and normal to the E-line respectively (Fig. 1b). The panoramic radiograph showed no pathology (Fig. 1 d). The patient had fair oral hygiene.

-Treatment Objectives

The treatment objectives were to 1) Improve patient’s oral hygiene habits, 2) Accept Class III skeletal relationship, 3) Resolve mandibular crowding with extraction of mandibular right lateral incisor, 4) Resolve bilateral posterior crossbite using a Hyrax expander with anterior sweep arms, 5) Resolve occlusal cant, 6) Normalize maxillary midline discrepancy, 7) Monitor periodontal health and refer to periodontist as needed, 8) Improve gingival display on smiling, and 9) Achieve consonant smile arc.

-Treatment Alternative

The alternative treatment plan was a surgical option with extraction of maxillary first premolars, and mandibular second premolars. Patient would be referred for surgery to accomplish a two-piece LeFort 1 maxillary expansion, posterior impaction, and mandibular bilateral sagittal split osteotomy setback surgery. Interproximal reduction as needed to reduce black triangles and manage Bolton discrepancy. Patient declined the surgical treatment option.

-Treatment Progress

Single-phase comprehensive treatment with extraction of mandibular right lateral incisor and Hyrax expander with sweep arms was performed. American Orthodontics (AO) 0.022 twin brackets were used. The overall active treatment lasted 44 months. We delivered in-house fabricated trays for 6 weeks post treatment for minor bite settling, followed by retention with maxillary and mandibular essix retainers.

Following sequences of treatment were delivered.

0.022 pre-adjusted brackets (AO Twin), MBT prescription were used. Oral hygiene instructions were reinforced to the patient throughout treatment. 1) Hyrax expander with sweep arms was cemented on the maxillary 4s and 6s with instruction to perform one turn every other day for eight weeks (being observed every 2 weeks). Once the first expander expanded to the fullest, we delivered a second expander with instruction to turn once every other day for 4 more weeks. Expansion was prescribed until the posterior crossbite was overcorrected bilaterally. 84 total turns were done for 21mm of expansion. The expander was stabilized for 7 months. 2) After expansion, the patient was referred for extraction of mandibular right lateral incisor. 3) We bonded maxillary 7-7 and mandibular 7-7. The maxillary archwires used included 0.012” Niti, 0.014” Niti, 18x18 Bioforce, 20x20 Bioforce, 0.014x0.025” CuNiTi, 0.016SS, 0.018SS, 16x22SS, 17x25SS. The mandibular archwires used included 0.012” Niti, 0.014” Niti, 0.018” Niti, 0.018SS, 16x22SS, 17x25SS, 19x25SS, 19x25TMA. 4) Elastomeric power chains were used to close the extraction space in the mandibular arch. 5) Root torquing spring was placed on the mandibular right canine to achieve lingual root torque. 6) 3/16th medium and heavy elastics were used on the left side to maintain crossbite correction. 7) IPR was performed on the mandibular 3-3 to increase overjet. 8) Finishing bends were used to detail and finish the case. 9) Patient was scanned for debond. Two mandibular aligners, and three maxillary aligners were made in ULab to settle bite in retention. Each aligner tray was worn for 2 weeks each. 1mm essix retainers were made for final retention in both arches. Post treatment records were taken after debonding.

-Treatment Results

**Figure 2:**
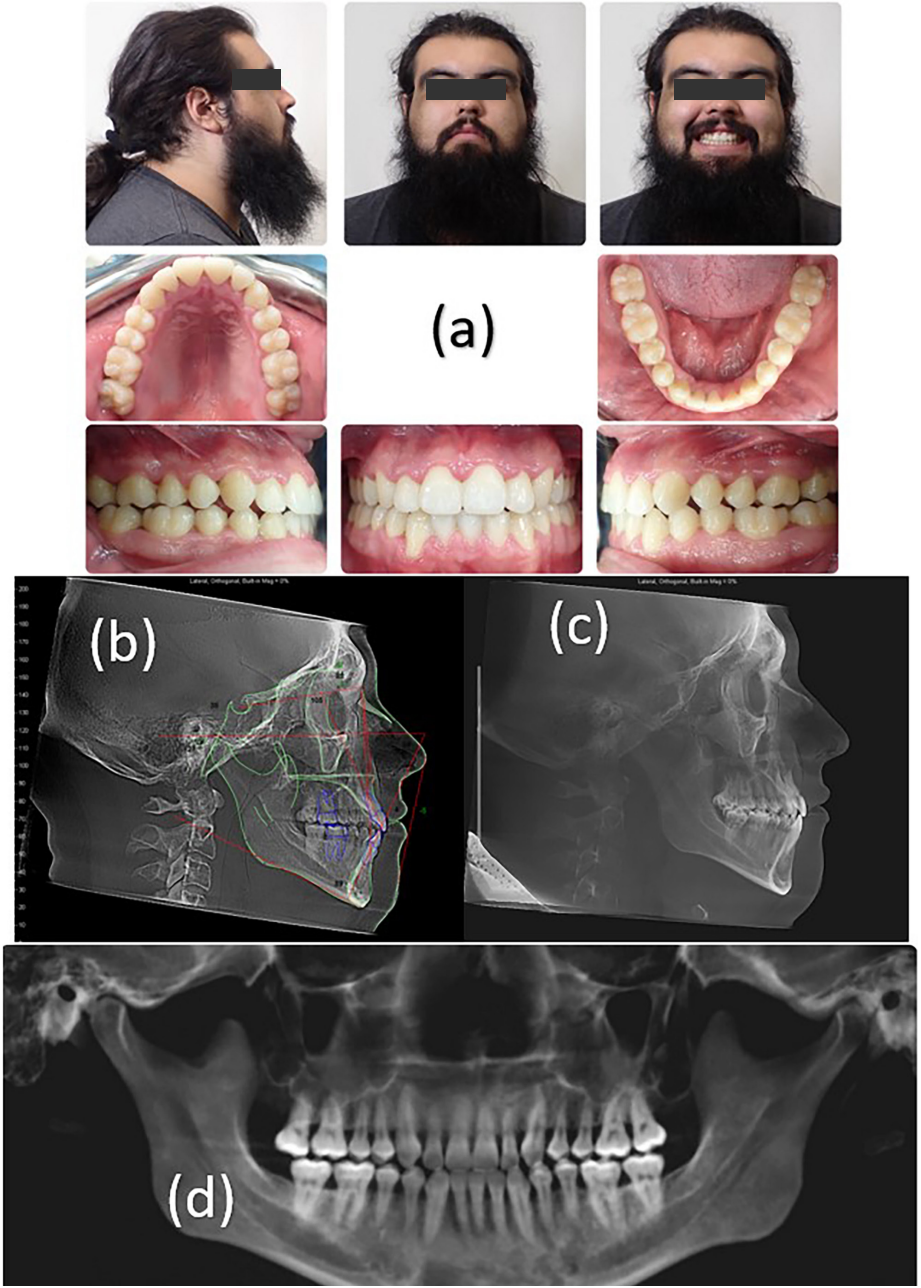
(a) Post treatment facial and intraoral photographs, (b) Post treatment cephalogram, (c) tracing and (d) panoramic radiograph.

**Figure 3:**
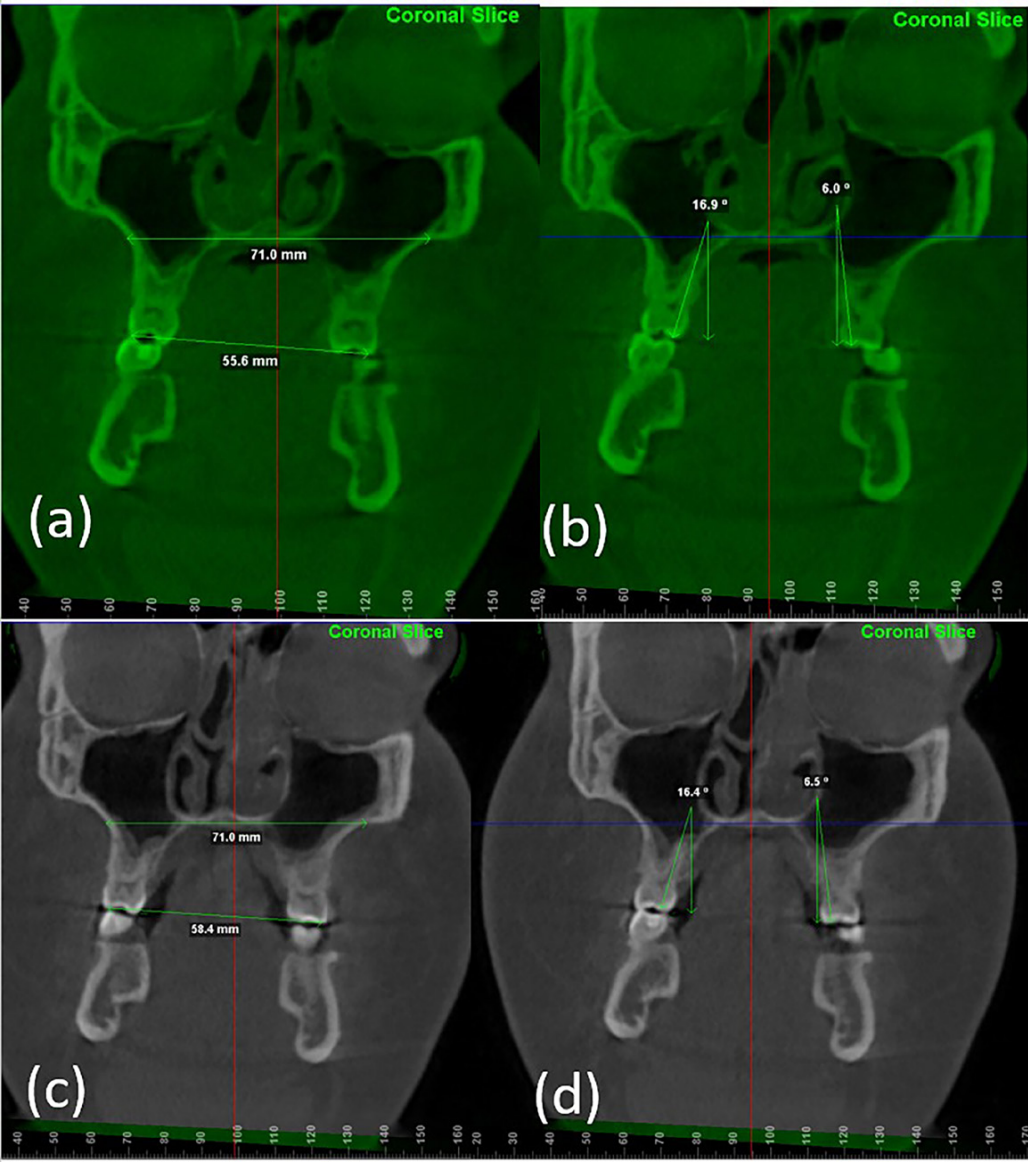
(a) Cone Beam Computed Tomography (CBCT) Face 2019: Coronal view. Inter-maxillary width. The intermaxillary width was maintained at 71.0mm. This measurement was taken from the outer cortex of the maxilla from left to right tangent to the hard palate, (b) CBCT Face 2019: Coronal view. The molar inclination measuring a line from palatal cusp going through the palatal root with a line perpendicular to the hard palate, (c) CBCT Face 2023: Coronal view. Inter-maxillary width maintained at 71.0mm. This measurement was taken from the outer cortex of the maxilla from left to right tangent to the hard palate superimposed with (a) to ensure measurement was taken in same plane of space. Improvement in the crossbite was achieved with the intermolar width changing from 55.6 mm to 58.4 mm, (d) CBCT Face 2023: Coronal view. The molar inclination measuring a line from palatal cusp going through the palatal root with a line perpendicular to the hard palate superimposed with (b) to ensure measurement was taken in same plane of space. The left maxillary first molar tipped buccally from 6.0° to 6.5° as expected. The right maxillary first molar decreased in angulation from 16.9° to 16.4°. Since there was improvement in the crossbite with the intermolar width changing from 55.6mm to 58.4 mm, the decrease in angulation post treatment was probably due to the torque built into the bracket system.

The post treatment records indicate that most of the treatment objectives were achieved. Patient’s oral hygiene was improved, CBCT measurement of patient’s airway indicated that the airway increased at the cross-sectional volume from 40 mm2 pre-treatment to 48 mm2 post-treatment. Class III occlusion was maintained for both right and left molars and canines. Maxillary and mandibular crowding was resolved with the extraction of the mandibular right lateral incisor (Fig. 2). The bilateral posterior crossbite was highly improved from pre-treatment showing an intermolar width changed from 55.6 mm to 58.4 mm. The molar inclination (measuring a line from palatal cusp going through the palatal root with a line perpendicular to the hard palate) on the right side changed from 16.9° to 16.4° and the left side molar inclination changed from 6.0° to 6.5°. The intermaxillary width stayed the same at 71.0 mm. (Reference (Fig. 3). Occlusal cant was resolved, and maxillary midline was placed to bisect the mandibular middle incisor. Pre and post TMJ cuts show no appreciable changes after treatment with no symptoms developing. Gingival display increased to 2mm. The mandibular plane rotated downward and backward from an FMA of 24.2° to 26.5°. The maxillary incisors were held at the same proclination (U1-SN, from 105.3° to 105.2°). The mandibular incisors were proclined (IMPA, from 84.4° to 89.2°) (Fig. 2b). Overbite stayed the same at 2mm. In terms of facial esthetics, no significant facial change was noticed.

## Discussion

Slow maxillary expansion in adults can be considered a viable treatment alternative for patients who do not want to undergo surgery or MARPE if they fit a certain criterion. In evaluating this case it was noted that the patient’s maxillary molars were lingually inclined and in posterior crossbite. This made the patient an ideal case for SME because the inclination of the teeth will allow for up-righting with a hyrax expander, understanding it is very unlikely to achieve skeletal expansion. A hyrax in this case is a good appliance choice because any effect provided by the appliance whether dental or skeletal is helpful in achieving the treatment objectives. After treatment with AO 0.022 twin brackets, the intermaxillary width was maintained based on measurement from superimposed CBCTs (Fig. 3a,c). This measurement was taken from the outer cortex of the maxilla from left to right tangent to the hard palate (8). This measured to be 71.0 mm. This indicated that there was no skeletal expansion, and all the expansion achieved was dentoalveolar. We measured the molar angulation with the angle between a line passing through the palatal cusp tip and palatal root apex and the vertical line perpendicular to the hard palate measured on the maxillary first molars in the coronal section (8). On the left maxillary first molar, buccal tipping was observed going from 6.0° to 6.5° as expected (Fig. 3b,d). On the right maxillary first molar, there was a decrease in angulation from 16.9° to 16.4° (Fig. 3b,d)). Since there was improvement in the crossbite with the intermolar width changing from 55.6 mm to 58.4 mm, the decrease in angulation post treatment was probably due to the torque built into the bracket system. The slow expansion initially tipped the tooth buccally, and the torque in the bracket helped upright the root during treatment. Even with overcorrection and retention with the Hyrax expander for 7 months, we noticed posterior relapse resulting in an edge-to-edge occlusion. At all phases of treatment to prevent relapse, we employed mechanics to maintain posterior overjet and prevent total relapse into crossbite. This included expansion of each maxillary arch wire to a broader arch form, and crossbite elastics. With the extraction of the mandibular incisor to camouflage the class III skeletal pattern and SME the final occlusal result was accepTable despite the patient declining the surgical treatment option. One of the limitations of this study was that we were unable to measure any relapse long term in the maxillary transverse dimension as the patient did not report back to the orthodontic clinic one-year post-debonding.

## Conclusions

The treatment goals for this adult patient were to correct the posterior crossbite and improve the malocclusion. There are many different surgical treatment options to achieve this result, but slow maxillary expansion was the least invasive and most agreeable to the patient. This case report shows that slow maxillary expansion can be used in an adult patient to achieve satisfactory results meeting the objectives set by both the patient and orthodontist.

## Data Availability

The datasets used and/or analyzed during the current study are available from the corresponding author.
